# Utilization of psychiatric care and antidepressants among people with different severity of depression: a population-based cohort study in Stockholm, Sweden

**DOI:** 10.1007/s00127-018-1515-0

**Published:** 2018-04-12

**Authors:** Yuying Sun, Jette Möller, Andreas Lundin, Samuel Y. S. Wong, Benjamin H. K. Yip, Yvonne Forsell

**Affiliations:** 1 School of Public Health, The University of Hong Kong, Hong Kong, China; 2 Epidemiology and Public Health Intervention Research group (EPHIR), Department of Public Health Sciences, Karolinska Institutet, Widerströmska huset, 3rd floor, Tomtebodav 18A, 111 77 Stockholm, Sweden; 3 The Jockey Club School of Public Health and Primary Care, The Chinese University of Hong Kong, Hong Kong, China

**Keywords:** Depression, Psychiatric care, Antidepressants, Outpatient visit, Hospitalization

## Abstract

**Purpose:**

To identify how severity of depression predicts future utilization of psychiatric care and antidepressants.

**Methods:**

Data derived from a longitudinal population-based study in Stockholm, Sweden, include 10443 participants aged 20–64 years. Depression was assessed by Major Depression Inventory and divided into subsyndromal, mild, moderate and severe depression. Outcomes were the first time of hospitalization, specialized outpatient care and prescribed drugs obtained from national register records. The association between severity of depression and outcomes was tested by Cox regression analysis, after adjusting for gender, psychiatric treatment history and socio-environmental factors.

**Results:**

The cumulative incidences of hospitalizations, outpatient care and antidepressants were 4.0, 11.2, and 21.9% respectively. Compared to the non-depressed group, people with different severity of depression (subsyndromal, mild, moderate and severe depression) all had significantly higher risk of all three psychiatric services (all log-rank test *P* < 0.001). Use of psychiatric care and antidepressants increased by rising severity of depression. Although the associations between severity of depression and psychiatric services were significant, the dose relationship was not present in people with previous psychiatric history or after adjusting for gender and other factors.

**Conclusions:**

People with subsyndromal to severe depression all have increased future psychiatric service utilization compared to non-depressed people.

**Electronic supplementary material:**

The online version of this article (10.1007/s00127-018-1515-0) contains supplementary material, which is available to authorized users.

## Introduction

The reported 12-month prevalence of DSM-IV (Diagnostic and Statistical Manual of Mental Disorders, 4th Edition) major depressive episodes in 18 countries ranges from 2.2 to 10.4% [1]. However, depressive disorders include a spectrum of severity [2] and the 12-month prevalence of any type of depression is higher, 13% according to the World Health Survey by World Health Organization [3]. There is a consensus that major depressive episodes always need treatment but there are also suggestions that people with persistent subthreshold depressive symptoms or mild to moderate depression should be treated [4]. The treatment recommendations include psychosocial interventions and physical activity and that pharmacological treatment with antidepressants should not be used for people without chronic physical health problems [4]. Antidepressants can be considered for people with subthreshold depressive symptoms that have been present for a long period (typically at least 2 years) or mild depression that persists after other interventions have failed or there is a past history of moderate or severe depression. However, two-thirds remain untreated since clinicians have been found to have considerable difficulty accurately identifying mild depression [5].

Subsyndromal depression is associated with limitations in psychological as well as physical functioning [6], but few studies have investigated the psychiatric service utilization. While one study showed that health care utilization increased by severity of depressive disorder [7], another reported increased utilization of psychiatric care for moderate to severe depression but not for mild depression [8]. A third study found increased utilization of health care or use of antidepressants for major but not minor depression after controlling for other factors [9]. Other predictors may also affect psychiatric service utilization. Wagner et al. [10] found that gender, race and level of education were all associated with mental health clinic visits (mental health specialist or to a family medicine provider) while negative life events and social support had no such association. Some studies have moreover indicated that life events and peer support, respectively, increased and decreased the likelihood of psychiatric hospitalizations [11, 12]. The current evidence is, however, inconclusive and there is a lack of studies that examine social and environmental factors together to predict psychiatric service utilization.

## Aim of the study

This study will use a population-based survey in Sweden that includes depression severity scales, demographic characteristics, childhood adversities, social network, coping style, stressful life events as well as psychiatric history, which have been linked to register-based data on hospitalization from Year 1998 to 2014, outpatient visits from Year 2001 to 2014 and prescribed medication records from Year 2005 to 2014. To provide further understanding of the psychiatric service utilization among people with different severity of depression, we will answer the following research questions: (1) What is the risk of hospitalizations, outpatient visit, and antidepressants treatment among people with different severity of depression? (2) How do socio-environmental factors affect the association between psychiatric services utilization and severity of depression? The rich datasets and long-term follow-up period, a wide range of socio-demographic and psychometric measurements will provide a more robust evidence to the current literature.

## Methods

This is a retrospective cohort study making use of register-based data linking to inpatient, outpatient and prescribed medication records.

### Participants

The subjects derived from the baseline examination of a longitudinal study in Stockholm County (PART, In Swedish: Psykisk hälsa, Arbete och RelaTioner; In English: the Mental health, Work, and Relations study). The study included three waves between 1998 and 2010, focused on mental health, work and relations among adult people residing in the Stockholm County, Sweden. The baseline sample comprised of people aged 20–64 years living in Stockholm County in the national population register (simple random sample). The self-reported questionnaires were sent by mail from 1998 to 2000. A total of 10,443 individuals responded to the questionnaire (response rate 53%). The characteristics of this population and non-response analysis were previously reported [13]. Non-response analysis demonstrated that participation was related to female gender, higher age, higher income and education, being born in the Nordic countries, and having no psychiatric diagnosis in the hospital discharge register or in the early retirement register [13]. All participants have provided informed consents. Ethical approval was received from the Karolinska Institutet, Stockholm ethical review board. The survey included questions of demographic characteristics, childhood adversities, social network, coping and stressful life events during the past 12 months as well as psychiatric rating scales. Through the participants’ 10-digit personal identity number, linkages were made to the National Patient Register (all inpatient records from 1998 to 2014 and outpatient records from 2001 to 2014) and the National Prescribed drug register (all dispensed prescribed drugs from 2005 to 2014), which were all held at the Social Board of Health and Welfare.

### Severity of depression

Depression was assessed using the Major Depression Inventory (MDI) [14, 15] from which diagnosis according to Diagnostic and Statistical Manual of Mental Diseases Fourth edition (DSM-IV), International Classification of Diseases and Related Health Problems 10th edition (ICD-10) as well as cut-off scores for different severity can be assessed. At first, the MDI score was used to classify participants according to depression severity [16, 17]. The participants were classified into four groups: ‘no depression or subsyndromal depression’ (score 0–20), ‘mild depression’ (score 21–25), ‘moderate depression’ (score 26–30) and ‘severe depression’ (score 31 or above). Those who had MDI scores of 20 or less, were further classified into ‘no depression’ and ‘subsyndromal depression’. The definition of subsyndromal depression was adopted from Judd et al. [18]: having at least two or more current depressive symptoms, present every day for most or all of the time, at least two weeks in duration. People who did not meet the criteria of subsyndromal depression were defined as ‘no depression’. Finally, five groups were generated by different severity of depression.

### Outcomes

Three indicators of mental health service utilization were used: (1) hospitalizations, (2) specialized outpatient care and, (3) medical treatment with antidepressant drugs. Hospitalizations (Inpatient records from 1998 to 2014) and specialized outpatient care (2001–2014) for mental disorders were based on ICD-10 chapter five Mental, Behavioral and Neuro-developmental disorders (F00–F99). Antidepressants usage (2005 to 2014 prescribed drugs register) was based on Anatomical Therapeutic Chemical (ATC) codes N05AN01 (Lithium) and N06A (Antidepressants). The dates of first incidences of these three outcomes were extracted from register data, including the admission date of hospitalizations, the treatment date of specialized care and dispensation date of antidepressants.

### Predictors

Age, gender, education level (primary, secondary, higher) and self-reported psychiatric disorder treatment history (previously diagnosed or treated for psychiatric disorder) were all included in the analysis. Twenty-three potentially negative life events (e.g. divorce, death of a child, financial strain) were assessed in the self-reported questionnaire which was explained in detail elsewhere [19]. The total number of negative life events was calculated and used as a continuous variable. Childhood adversities were assessed by two questions about death of parents (dichotomous) and disturbances in the family (no, small and/or short periods, severe and/or long periods) during childhood (before the age of 18 years). Coping strategies when facing problems and difficulties in life were measured by an instrument developed by Aronsson and Stromberg [20]. The instrument included 12 items which were rated as ‘yes’ or ‘no’ answers. Negative coping (five questions: get irritated and upset, feeling helpless, get bad conscious, worry, wait and hope problems being solved naturally) and positive coping (seven questions: put more energy to fix problems, able to relax to face problems, view the situation as a potential opportunity for changing, seek support and help, seek information to solve problem, try to organize and plan life better, feel relaxed) were separated as two independent factors in the analysis. Availability of social attachment (ASAT) was assessed with three questions from a Swedish modification of the Interview Schedule for Social Interaction (ISSI) [21, 22]. The nine items which comprise the availability dimension consisted of two related categories; the availability of social integration and the availability of attachment. The availability of social integration (four items) was scored on a 6-point Likert scale ranging from 1 to 6. The availability of attachment (five items) was scored on a 4-point Likert scale, ranging from 1 to 4. The two subscales were summed into a total score ranging from 9 to 44, with higher scores indicating greater availability of social relationships.

### Statistical analysis

The time-to-event was defined as the interval between recruitment (the date of completing the questionnaire) and either the first date of the outcome occurrence or the end of follow-up (12/31/2014). Cumulative incidence rates were depicted by Kaplan–Meier curves. Differences between the incidence rates and linear trends between the exposure groups were compared by log-rank test. Cox regression analysis was used to examine the association between severity of depression and hospitalization, specialized outpatient visits and antidepressants. We reported hazard ratio (HR) and 95% asymptotic confidence intervals (CI) as measures of incidence rate ratios. Individuals with missing data were deleted from all analyses. 1.3% of the participants had missing items, leaving 10,304 (98.7%) participants included in the final analyses. Statistical analysis was performed using SPSS 23.0 for Windows (SPSS Inc., Chicago, IL, USA).

## Results

### Characteristics

Table 1 shows the baseline characteristics of the study population, stratified according to depression severity. No depression was present in 77.1% of the participants (*n* = 7943), 15.2% (*n* = 1562) had subsyndromal depression, 3.4% (*n* = 346) had mild depression, 1.9% (*n* = 196) had moderate depression and 2.5% (*n* = 257) had severe depression.

**Table 1:** Characteristics of participants at baseline and incidence of outcomes

Characteristics	Depression					
	Total *N* = 10,304	No *n* = 7943	Subsyndromal *n* = 1562	Mild *n* = 346	Moderate *n* = 196	Severe *n* = 257
Age, Mean (SD)	41.4 (12.5)	42.1 (12.5)	38.2 (12.2)	40.1 (12.2)	39.1 (12.0)	42.1 (11.2)
Gender, *n* (%)						
Male	4600 (44.6)	3785 (47.7)	557 (35.7)	102 (29.5)	61 (31.1)	95 (37.0)
Female	5704 (55.4)	4158 (52.3)	1005 (64.3)	244 (70.5)	135 (68.9)	162 (63.0)
Education level, *n* (%)						
Primary	1500 (14.6)	1086 (13.7)	238 (15.3)	61 (17.7)	41 (21.0)	74 (29.2)
Secondary	4300 (41.9)	3300 (41.7)	673 (43.2)	152 (44.1)	83 (42.6)	92 (36.4)
Higher level	4473 (43.5)	3535 (44.6)	648 (41.6)	132 (38.3)	71 (36.4)	87 (34.4)
Parental death, *n* (%)	542 (5.3)	388 (4.9)	90 (5.8)	22 (6.4)	22 (11.2)	20 (7.8)
Familial hassles, *n* (%)						
No	6600 (64.2)	5415 (68.3)	830 (53.3)	170 (49.3)	80 (41.0)	105 (41.3)
Small	2431 (23.6)	1756 (22.1)	449 (28.8)	96 (27.8)	58 (29.7)	72 (28.3)
Severe	1249 (12.1)	758 (9.6)	278 (17.9)	79 (22.9)	57 (29.2)	77 (30.3)
Negative coping, Mean (SD)	2.2 (1.4)	1.9 (1.3)	2.8 (1.3)	3.2 (1.3)	3.2 (1.3)	3.6 (1.3)
Positive coping, Mean (SD)	4.9 (1.5)	5.1 (1.4)	4.4 (1.6)	3.8 (1.8)	3.7 (1.6)	3.4 (1.6)
Social network, Mean (SD)	27.3 (3.9)	27.3 (3.9)	27.0 (4.0)	26.9 (4.5)	27.2 (4.4)	27.0 (4.3)
Negative life events, Mean (SD)	1.7 (1.7)	1.4 (1.5)	2.4 (1.8)	2.8 (2.0)	3.1 (2.1)	3.9 (2.5)
Psychiatric treatment history, *n* (%)	629 (6.1)	333 (4.2)	161 (10.3)	53 (15.3)	27 (13.8)	55 (21.4)
Cumulative incidence, *n* (%)						
Hospitalizations	416 (4.0)	225 (2.8)	98 (6.3)	36 (10.4)	16 (8.2)	41 (16.0)
Outpatient care	1153 (11.2)	623 (7.8)	292 (18.7)	90 (26.0)	59 (30.1)	94 (36.6)
Antidepressants	2258 (21.9)	1354 (17.0)	539 (34.5)	143 (41.3)	89 (45.4)	133 (51.8)
Incident rate per 1000 person-years						
Hospitalizations	2.73	1.90	4.31	7.37	5.77	11.88
Outpatient care	7.73	5.34	13.30	19.57	22.57	28.88
Antidepressants	16.03	12.12	27.20	34.32	38.10	46.15

*SD* standard deviation

### Cumulative incidence and incidence rates of outcomes

During the whole follow-up period, the cumulative incidences of hospitalizations, specialized outpatient care and antidepressants were 4.0, 11.2, and 21.9% respectively. Around a third of the people with subsyndromal depression had antidepressants between Year 2005 and 2014. More than 40% of those with mild to moderate depression and more than half of those with severe depression used antidepressants at least once during the follow-up period. The overall incidence rates of hospitalizations, outpatient visit and antidepressants were 2.7, 7.7 and 16.0 per 1000 person-years (Table 1). The overall incidence rates of hospitalizations were higher in those with subsyndromal depression (HR = 2.26, 95% CI 1.78–2.86), mild (HR = 3.83, 95% CI 2.69–5.45) to moderate (HR = 3.00, 95% CI 1.81–4.99) and severe depression (HR = 6.13, 95% CI 4.40–8.55) compared with those who were non-depressed. Individuals with subsyndromal to severe depression (subsyndromal: HR = 2.54, 95% CI 2.21–2.92; mild: HR = 3.77, 95% CI 3.03–4.71; moderate: HR = 4.34, 95% CI 3.32–5.66; severe: HR = 5.65, 95% CI 4.54–7.01) had a higher risk of outpatient care than those who were non-depressed. Individuals with subsyndromal to severe depression (subsyndromal: HR = 2.27, 95% CI 2.06–2.51; mild: HR = 2.88, 95% CI 2.42–3.42; moderate: HR = 3.19, 95% CI 2.58–3.96; severe: HR = 3.93, 95% CI 3.29–4.70) had a higher risk of using antidepressants than those who were non-depressed.

**Fig. 1:**
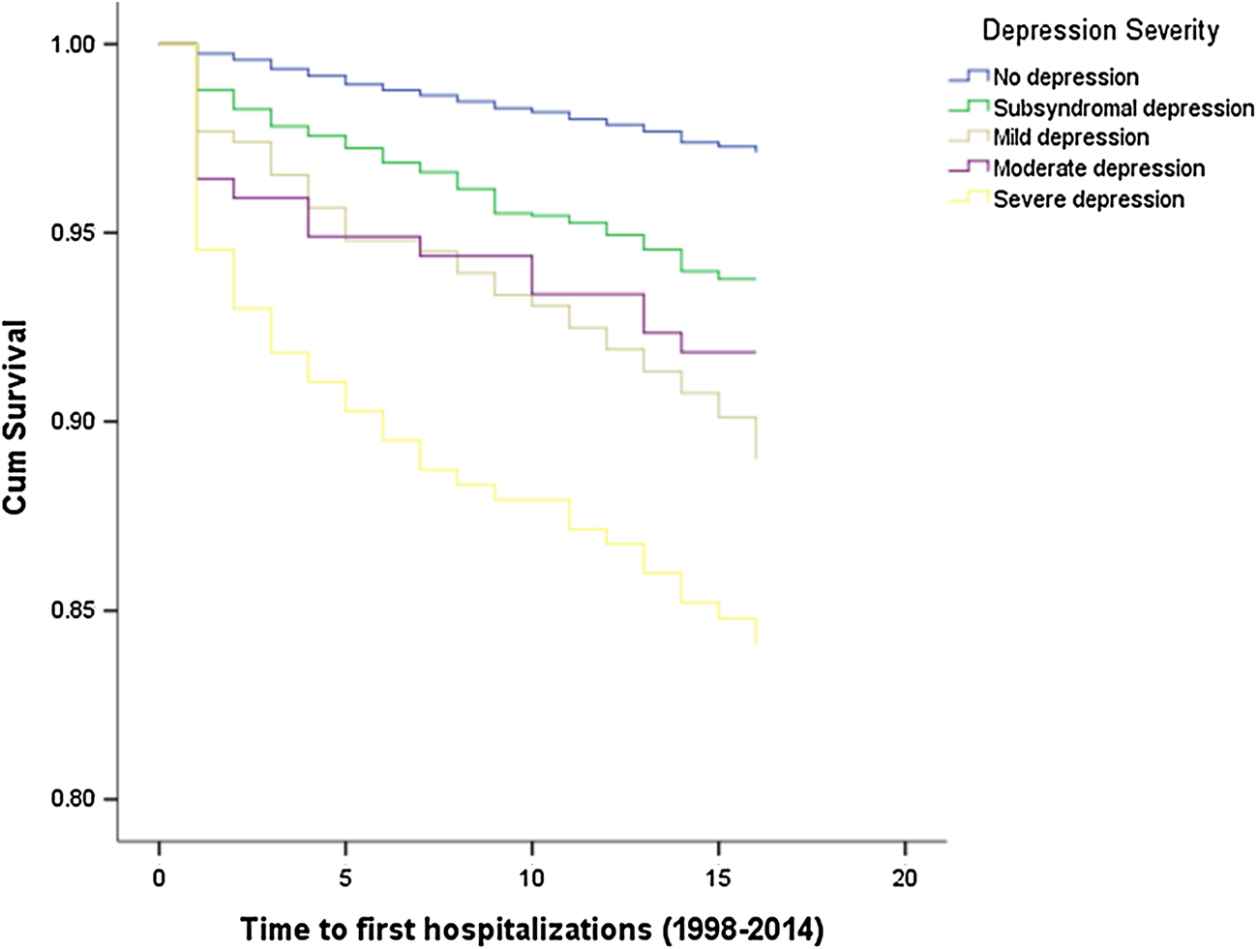
The time to first hospitalization among people with different severity of depression (1998–2014)

**Fig. 2:**
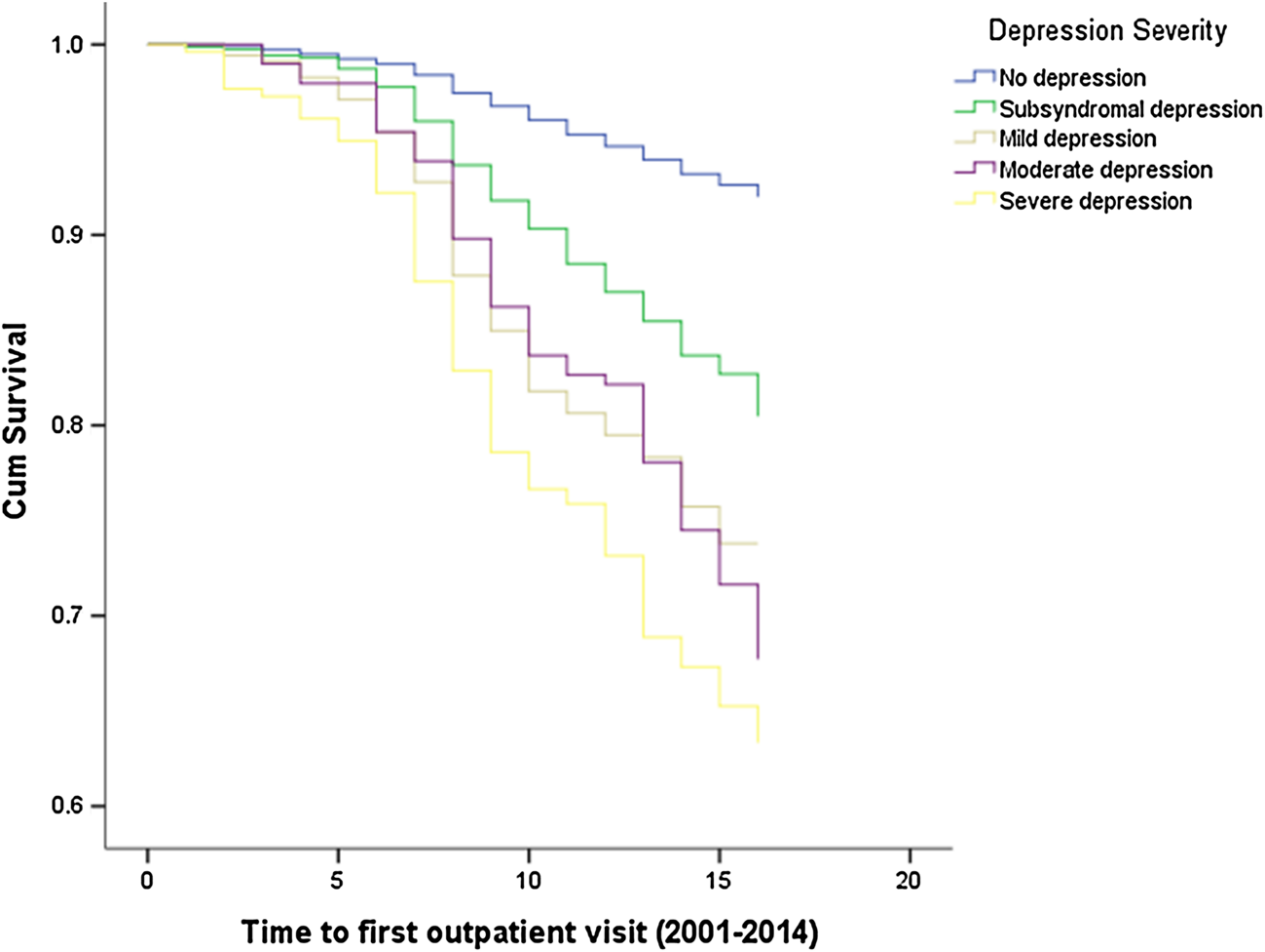
The time to first outpatient visit among people with different severity of depression (2001–2014)

**Fig. 3:**
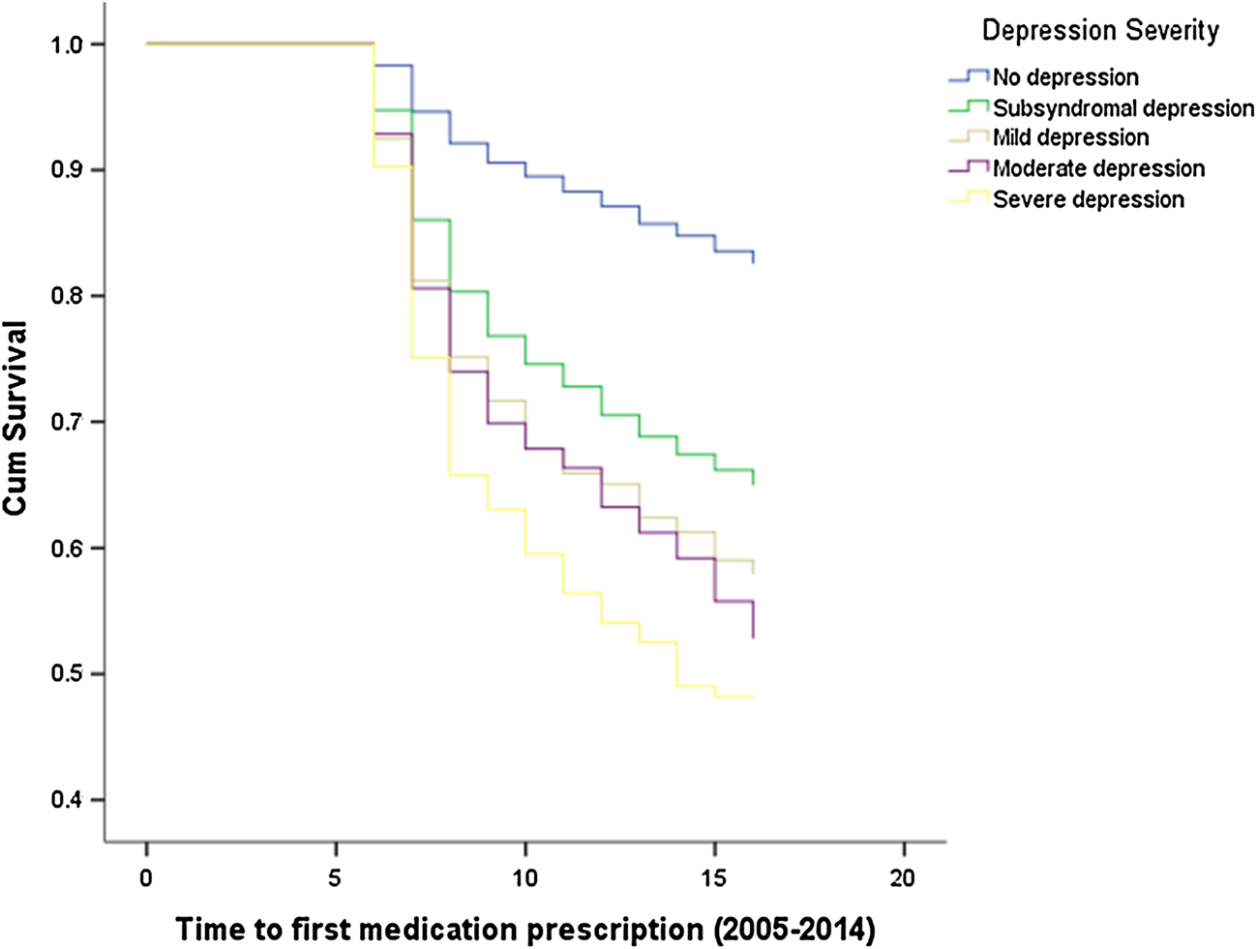
The time to first medication prescription among people with different severity of depression (2005–2014)

Figures 1, 2, 3 illustrate the results of time-to-event analyses among people with different severity of depression. Pairwise comparisons revealed that the non-depressed group had consistently lower risk of using all three psychiatric services compared to the other groups (all log-rank test *P* < 0.001). People with subsyndromal depression had significantly lower risk of hospitalizations compared to those with mild (*χ*^2^=7.51, *P* = 0.006) or severe depression (*χ*^2^=31.17, *P* < 0.001), and lower risk of outpatient visits compared to those with mild (*χ*^2^=11.20, *P* = 0.001) or moderate to severe depression (*χ*^2^=14.90, 48.64, respectively, all *P* < 0.001). Risk of antidepressants usage among people with subsyndromal depression was also significantly lower than that for mild (*χ*^2^=6.51, *P* = 0.011), moderate (*χ*^2^=9.20, *P* = 0.002) and severe depression (*χ*^2^=33.44, *P* < 0.001). People with mild depression had significantly lower risk of hospitalizations (*χ*^2^=4.23, *P* = 0.040), outpatient visits (*χ*^2^=7.66, *P* = 0.006) and antidepressants (χ^2^=6.86, P = 0.009) compared to those with severe depression. People with moderate depression had a lower risk of hospitalizations (*χ*^2^=6.00, *P* = 0.014) compared to those with severe depression but there were no differences in outpatient visits (*χ*^2^=2.54, *P* = 0.111) or antidepressants usage (*χ*^2^=2.40, *P* = 0.122).

Cox regression analysis demonstrated that in both males and females, the risk of hospitalizations, outpatient visits and antidepressants use were increased by severity of depression (Table 2). Stratifying by previous psychiatric history showed that the dose relationship between depression severity and hospitalization, outpatient visits or antidepressants was stronger for those people who had never been treated previously. No apparent risk increase for hospitalization across depression severity was found for those with psychiatric history.

**Table 2:** Cox regression analysis of psychiatric service utilization among people with different severity of depression (*N* = 10,304)

Predictors	Hospitalizations HR (95% CI)		Outpatient care HR (95% CI)		Antidepressants HR (95% CI)	
Gender	Male	Female	Male	Female	Male	Female
No depression	1.00 (reference)	1.00 (reference)	1.00 (reference)	1.00 (reference)	1.00 (reference)	1.00 (reference)
Subsyndromal	2.48 (1.76,3.49)**	2.25 (1.61,3.13)**	2.34 (1.86,2.94)**	2.65 (2.22,3.16)**	2.26 (1.88,2.72)**	2.11 (1.87,2.38)**
Mild	3.44 (1.86,6.37)**	4.40 (2.84,6.82)**	4.40 (3.04,6.37)**	3.51 (2.66,4.63)**	3.14 (2.24,4.41)**	2.52 (2.06,3.08)**
Moderate	2.08 (0.77,5.64)	3.82 (2.10,6.95)**	4.19 (2.60,6.74)**	4.40 (3.18,6.09)**	2.69 (1.70,4.25)**	3.07 (2.41,3.92)**
Severe	7.32 (4.57,11.74)**	5.67 (3.54,9.08)**	5.49 (3.85,7.84)**	5.73 (4.35,7.55)**	4.85 (3.58,6.56)**	3.33 (2.67,4.15)**
Psychiatric history	No	Yes	No	Yes	No	Yes
No depression	1.00 (reference)	1.00 (reference)	1.00 (reference)	1.00 (reference)	1.00 (reference)	1.00 (reference)
Subsyndromal	2.17 (1.65,2.84)**	1.34 (0.81,2.22)	2.46 (2.11,2.87)**	1.61 (1.15,2.26)**	2.19 (1.96,2.44)**	1.67 (1.29,2.16)**
Mild	3.01 (1.92,4.72)**	2.61 (1.44,4.73)**	3.45 (2.67,4.47)**	2.35 (1.50,3.69)**	2.70 (2.22,3.28)**	1.88 (1.29,2.74)**
Moderate	3.30 (1.88,5.78)**	0.94 (0.29,3.03)	4.12 (3.03,5.60)**	2.41 (1.38,4.18)**	3.10 (2.44,3.95)**	1.83 (1.13,2.96)*
Severe	6.38 (4.31,9.43)**	1.96 (1.03,3.74)*	5.45 (4.22,7.04)**	2.49 (1.62,3.84)**	3.81 (3.10,4.69)**	1.87 (1.30,2.69)**
All the included factors	Crude	Adjusted	Crude	Adjusted	Crude	Adjusted
No depression	1.00 (reference)	1.00 (reference)	1.00 (reference)	1.00 (reference)	1.00 (reference)	1.00 (reference)
Subsyndromal	2.26 (1.78,2.86)**	1.63 (1.26,2.11)**	2.54 (2.21,2.92)**	1.85 (1.59,2.16)**	2.27 (2.06,2.51)**	1.76 (1.58,1.96)**
Mild	3.83 (2.69,5.45)**	2.42 (1.66,3.53)**	3.77 (3.03,4.71)**	2.50 (1.98,3.16)**	2.88 (2.42,3.42)**	1.86 (1.55,2.23)**
Moderate	3.00 (1.81,4.99)**	1.68 (0.99,2.86)	4.34 (3.32,5.66)**	2.65 (2.00,3.53)**	3.19 (2.58,3.96)**	2.02 (1.61,2.53)**
Severe	6.13 (4.40,8.55)**	2.46 (1.66,3.66)**	5.65 (4.54,7.01)**	2.85 (2.21,3.66)**	3.93 (3.29,4.70)**	2.06 (1.68,2.52)**
Gender (female)	–	0.67 (0.55,0.82)**	–	0.94 (0.84,1.07)	–	1.52 (1.39,1.67)**
Psychiatric history (yes)	–	3.07 (2.40,3.92)**	–	2.52 (2.14,2.95)**	–	2.26 (2.00,2.56)**
Education level(1)	–	0.64 (0.49,0.83)**	–	0.86 (0.72,1.02)	–	0.91 (0.82,1.04)
Education level(2)	–	0.57 (0.44,0.74)**	–	0.86 (0.72,1.02)	–	0.88 (0.78,0.99)*
Age	–	1.00 (0.99,1.01)	–	0.99 (0.986,0.996)**	–	1.01 (1.006,1.013)**
Parent death in childhood	–	1.04 (0.71,1.53)	–	0.99 (0.78,1.27)	–	1.05 (0.89,1.25)
Disturbances in family(1)	–	1.11 (0.87,1.42)	–	1.17 (1.02,1.35)*	–	1.19 (1.08,1.32)**
Disturbances in family(2)	–	1.75 (1.36,2.25)**	–	1.46 (1.25,1.71)**	–	1.33 (1.18,1.50)**
Negative Coping	–	1.04 (0.96,1.13)	–	1.08 (1.03,1.13)**	–	1.08 (1.05,1.12)**
Positive Coping	–	1.02 (0.95,1.09)	–	0.99 (0.95,1.03)	–	0.96 (0.93,0.99)**
Social network	–	0.95 (0.93,0.97)**	–	0.97 (0.95,0.98)**	–	0.98 (0.97,0.99)**
Negative Life events	–	1.15 (1.10,1.21)**	–	1.09 (1.06,1.12)**	–	1.05 (1.03,1.08)**

Education Level (1): Secondary level; Education Level (2): High level; Disturbances in family (1): small and/or short periods of disturbances in family during childhood; Disturbances in family (2): severe and/or long periods of disturbances in family during childhood

*HR* hazard ratio, *CI* confidence interval

*P < 0.05; **P < 0.01

The associations between severity of depression and hospitalizations, outpatient visit and antidepressants were all significant even after controlling for gender, psychiatric history, education level, age, childhood adversities, negative coping, positive coping, social network and negative life events. From the adjusted model, we found that the common factors associating with psychiatric care and antidepressants were previous psychiatric history, severe and/or long periods of disturbances in family during childhood, less availability of social attachment and more negative life events. Being male, low education level was also associated with the first hospitalization. Outpatient care was also associated with younger age, small or short periods of disturbances in family during childhood and negative coping. Being female, low education level, older age, small or short periods of disturbances in family during childhood, and negative coping were all associated with the first antidepressants taken.

## Discussion

The results showed that persons affected by subsyndromal to severe depression had higher risk of utilizing psychiatric care and antidepressants than non-depressed people. The risk of hospitalizations and outpatient visits were around twofold, threefold, sixfold higher in those with subsyndromal depression, mild to moderate and severe depression than those who were non-depressed. This could be due to the fact that people with subsyndromal or mild depression developed more severe symptoms over time. Depression has a fluctuation course [18, 23] and the incidence of major depressive disorder among people with subthreshold depression has been reported to be high (8–47%) [24–27]. It is also possible that individuals with mild to moderate depression were in recovery or remission stage from major depressive disorder at the time of the PART study. Some researchers have suggested that mild to moderate and severe depressive disorders should be considered as a continuum rather than a set of discrete subtypes [28, 29]. According to pairwise comparisons, severe depression was found to have higher risk of hospitalization when comparing to all the lower level severity of depression, but no difference was found in outpatient care and antidepressants use when comparing to moderate depression. This indicated that people with subsyndromal to mild depression might be in a transition stage between normal and moderate to severe depression, but can be treated in a different way with severe depression in practice due to their different impact in the long run.

We found that for those without a previous psychiatric history, there was a trend of increasing risk for psychiatric care and antidepressants usage by severity of depression. But for those with previous psychiatric history, the extent of future service utilization was similar among people with subsyndromal to severe depression.

Our results also showed that the associations between severity of depression were still significant after adjusting for other factors, although attenuated. The subsyndromal to moderate depression group had a hazard ratio for psychiatric care and medication usage similar to that for severely depressed. This implies that when facing patients with subsyndromal to severe depression in the clinic, their future psychiatric care and medication usage would be similar in the long run. A study based on heart failure patients also reported a similar hazard ratio for all-cause outpatient and inpatient services among those individuals with moderate to severe depression [8]. Our findings agreed with theirs, although we used a general population sample and looked specifically at psychiatric services. According to the fully adjusted model, the common factors associated with both psychiatric care and antidepressants use were previous psychiatric history, severe and/or long periods of disturbances in family during childhood, less availability of social attachment and more negative life events. It is worth mentioning that men had a higher risk of hospitalization but lower risk of taking antidepressants when comparing with women. Older people were less likely to use outpatient service but have a higher risk of taking antidepressants.

In this study, use of psychiatric care and antidepressants during 16-year follow-up of a general population cohort assessed for depression severity were examined using register-based data. Scientific evidence has complemented the current literature by utilizing a wide range of socio-demographic and psychometric measurements. However, the study has some limitations. First, the response rate to the postal questionnaires was only 53%, which limited the external comparison. However, previous studies using PART data demonstrated that the study participants can most likely be a base for generalizing risk indicators and social consequences of mental disorder to the general population [13]. Second, the postal MDI questionnaire was used to identify the severity of depression and this may be less accurate than a clinician’s diagnosis. However, clinicians have been found to have considerable difficulty accurately identifying less severe depression [5]. Comparison between MDI and psychiatric interview had generally shown that depression can be identified reliably with the scale [30], but any misclassification could have diluted or biased our associations. Third, the register of inpatient care was only complete from 1998 to 2014, and outpatient visits and drug register data were only available from 2001 to 2005, respectively. Thus the associations between severity of depression and outpatient visits or antidepressants might have been biased. But the direction is unclear since we do not know how the severity of depression and other socio-environmental factors fluctuated during our follow-up period. Fourth, service utilization and medication use were identified as the date of first inpatient, outpatient or medication record, meaning there was no measure of amount of service utilization. Our study only focused on the association between severity of depression and the first onset of the service utilization and medication use, but it might be interesting to see how severity of depression impacts the intensity of service utilization and medication use in the next step. Last, the 23 potentially negative life events were summed into a total score and were consequently given equal importance rather than being analyzed individually. This may have diluted the effect of a single specific and potent event, reflected in the positive, albeit weak, association between negative life events and hospitalization, outpatient care and antidepressants.

With a large sample size of a non-clinical population, and a 16-year follow-up, we conclude that people affected by subsyndromal to severe depression have higher utilization of psychiatric service than non-depressed people. For people with previous psychiatric history, the risk of future psychiatric service utilization was similar for all severity groups. The results highlight the importance of paying equal attention to people with depression regardless of severity although different actions should be taken.

## Electronic supplementary material

Below is the link to the electronic supplementary material.

Supplementary material 1 (DOCX 18 KB)
