# Supplementary material for: Utilization of psychiatric care and antidepressants among people with different severity of depression: a population-based cohort study in Stockholm, Sweden
Source: Soc Psychiatry Psychiatr Epidemiol. 2018 Apr 12;53(6):607–15. doi: 10.1007/s00127-018-1515-0 (PMC5959996; doi:10.1007/s00127-018-1515-0)
Supplement: Supplementary file 1 — Supplementary material 1 (DOCX 18 KB) [file 127_2018_1515_MOESM1_ESM.docx]

Supplementary Table 1. Estimated time (years) to first hospitalizations, outpatient visits and antidepressants use

| Severity of Depression | Estimated time to first hospitalizations  Mean(95%CI) | Estimated time to first outpatient visit  Mean(95%CI) | Estimated time to first antidepressants use  Mean(95%CI) |
| --- | --- | --- | --- |
| No depression | 15.75(15.71,15.78) | 15.48(15.44,15.53) | 14.78(14.71,14.84) |
| Subsyndromal | 15.37(15.23,15.50) | 14.75(14.60,14.90) | 13.24(13.03,13.44) |
| Mild | 14.98(14.62,15.34) | 13.99(13.59,14.39) | 12.61(12.14,13.07) |
| Moderate | 15.04(14.55,15.32) | 14.03(13.52,14.53) | 12.46(11.84,13.07) |
| Severe | 14.17(13.60,14.75) | 13.18(12.64,13.72) | 11.62(11.06,12.18) |

Note. CI: Confidence Interval.
